# Clinical Implications of Polymicrobial Synergism Effects on Antimicrobial Susceptibility

**DOI:** 10.3390/pathogens10020144

**Published:** 2021-02-01

**Authors:** William Little, Caroline Black, Allie Clinton Smith

**Affiliations:** 1 Department of Honors Studies, Texas Tech University, Lubbock, TX 79424, USA; william_little@outlook.com; 2 Department of Biological Sciences, Texas Tech University, Lubbock, TX 79424, USA; caroline.black@ttu.edu

**Keywords:** polymicrobial, antimicrobial susceptibility, synergism, interactions, clinical, diagnostics

## Abstract

With the development of next generation sequencing technologies in recent years, it has been demonstrated that many human infectious processes, including chronic wounds, cystic fibrosis, and otitis media, are associated with a polymicrobial burden. Research has also demonstrated that polymicrobial infections tend to be associated with treatment failure and worse patient prognoses. Despite the importance of the polymicrobial nature of many infection states, the current clinical standard for determining antimicrobial susceptibility in the clinical laboratory is exclusively performed on unimicrobial suspensions. There is a growing body of research demonstrating that microorganisms in a polymicrobial environment can synergize their activities associated with a variety of outcomes, including changes to their antimicrobial susceptibility through both resistance and tolerance mechanisms. This review highlights the current body of work describing polymicrobial synergism, both inter- and intra-kingdom, impacting antimicrobial susceptibility. Given the importance of polymicrobial synergism in the clinical environment, a new system of determining antimicrobial susceptibility from polymicrobial infections may significantly impact patient treatment and outcomes.

## 1. Introduction

With recent developments in sequencing technologies, it has been shown that many infections can be polymicrobial in nature, potentially leading to worse patient outcomes. Current routine clinical models, however, focus on unimicrobial culture-based methods to determine the causative agent. It is now well-known that many chronic infections are often polymicrobial in nature [1]. Often, chronic infections are difficult to treat as polymicrobial interactions can lead to decreased antibiotic efficacy [1]. Recently developed next generation sequencing (NGS) technologies have led to increased understanding of polymicrobial infections, as they have helped to eliminate culture-bias in microbial identification. Culture-based microbial identification is a common method used in clinical laboratories to identify the microorganisms in samples [2]. However, it has been shown that certain species of microbes are often lost in culture, either due to competition from other microorganisms, the need for specific growth requirements, or other causes. NGS has proven effective to accurately recount the composition of microbial communities that would normally take multiple culture attempts using different differential and/or selective media and non-routine culture methods [3]. With NGS helping to more accurately identify the microorganisms in samples, it is now being demonstrated that more infections are polymicrobial than was previously recognized. Studies examining the polymicrobial nature of infectious processes are discussed below, and unless otherwise noted, the cited studies determined the polymicrobial consortia utilizing NGS methods.

## 2. Many Infections Are Polymicrobial

There have been many studies that have shown that infections, particularly chronic infections, are often polymicrobial. Nair et al. has shown that *S. aureus* often co-infects with species such as *Haemophilus influenzae, Enterococcus faecalis, Pseudomonas aeruginosa, Streptococcus pneumoniae, Corynebacterium* sp., *Lactobacillus* sp., *Candida albicans*, and the influenza virus; *S. aureus* can work cooperatively with *C. albicans, E. faecalis, H. influenzae*, and the influenza virus [4]. After analyzing 36 *Citrobacter freundii* infections in a Taiwanese hospital, Liu et al. demonstrated that 15 (41.6%) were polymicrobial in nature [5]. Looking at *Streptococcus anginosus* group (SAG) infections in children in Tokyo (using culture-dependent methods), Furuichi and Horikoshi showed that 45 out of 52 (87%) of the patients had infections that were polymicrobial in nature; over 70% of *S. anginosus* and *S. constellatus* infections were also colonized by obligate anaerobes, with *Bacteroides* spp. most commonly associated with *S. anginosus* [6].

### 2.1. Skin and Soft Tissue Infections

In a cross-sectional study conducted by Jaju et al. of 125 diabetic foot ulcer (DFU) samples, 21 (16.80%) were polymicrobial in nature (based on culture-dependent methods) [7]. Wagner grade classification of these DFUs showed that polymicrobial infections had a higher patient morbidity, with 0% of polymicrobial infections being grade 1 and 27.20% of polymicrobial infections being grade 5 [7]. It was recently shown that necrotizing soft tissue infections (NSTIs), infamous for their high patient morbidity and mortality, contain several species of anaerobes, many of which are difficult to culture. Furthermore, 16s rRNA sequencing revealed a more diverse bacterial community than attempts at culturing displayed [8]. Bessa et al. collected samples from 217 chronically infected wounds which showed infections from 28 different species of bacteria. Of these infections, 59 (27.1%) were polymicrobial- mostly co-infections between *S. aureus* and *P. aeruginosa* [9]. Rhoads et al. compared microbial identification between culture-dependent and molecular methods in 51 samples, and recovered a mean of 1.8 ± 0.9 bacterial genera and 14.8 ± 7.5 bacterial genera using culture-based and molecular methods, respectively, demonstrating the polymicrobial nature of wounds [10]. An analysis of the chronic wounds of 2963 patients performed by Wolcott et al. also demonstrated that most chronic wounds are polymicrobial in nature, as only 7% of the microbiomes from the wound samples collected showed at least a 99%, if not greater, predominant single species present in the infection [11]. There is a significantly higher chance of recurring infection if a wound is polymicrobial, as shown by a study looking at prosthetic foot fungal infections co-infected with bacteria [12]. When analyzing chronic wounds, Dowd et al. found that many fungi, especially those belonging to the genus *Candida*, were present in polymicrobial infections;while *Candida* was the most common genus isolated, they also isolated fungi from the genera *Curvularia, Malessezia, Aureobasidium, Cladosporium, Ulocladium, Engodontium* and *Trichtophyton* from polymicrobial wound infections [13]. 

### 2.2. Respiratory

Looking at chronic infections in cystic fibrosis (CF) patients, Henson et al. discovered they are often due to a diverse community of pathogens, including viruses, bacteria, and fungi [12]. CF patients have been shown to have increased virulence factor production by *P. aeruginosa* when in a polymicrobial environment with streptococci; streptococci growth is also increased in the presence of *P. aeruginosa* [14].

### 2.3. Otolaryngology

By assessing abscesses in endodontics, Segura-Egea et al. found that 98% of the 94 patients in the study had infections that were polymicrobial [15]. Using MALDI-TOF and multiplex PCR, Uddén et al. found that many of the chronic suppurative otitis media (CSOM) infections in the middle ear were polymicrobial [16]. When Dunne et al. looked at infections in the nasopharynx, *M. catarrhalis* showed a positive association with both *S. pneumoniae* (Odds Ratio (OR) 3.07, CI 1.91–4.94) and *H. influenzae* (OR 2.34, CI 1.40–3.91) [17]. 

The more accurately clinical microbiologists can identify the causative agent of infection, the better treatment the patient will receive, potentially leading to better patient outcomes. With so many studies demonstrating that certain infections are often polymicrobial in nature, the use of monocultures in the diagnostic setting may not generate results representative or comprehensive of the clinical environment; this methodology should be reconsidered in an effort to help increase the chances for better patient outcomes. The current methodology employed to determine antimicrobial susceptibility of most samples in the majority of clinical laboratories is described further. 

## 3. Clinical Determination of Antimicrobial Susceptibility

**Figure 1:**
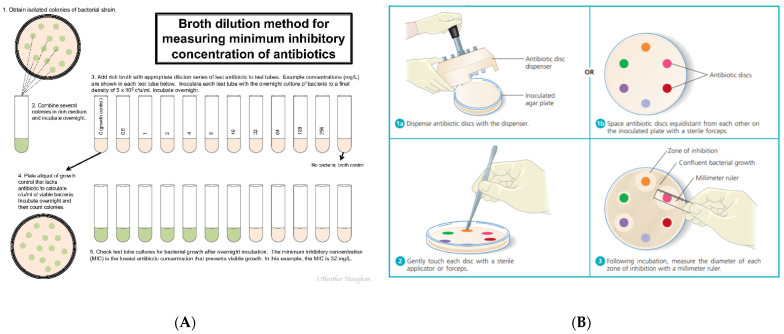
A schematic of two minimum inhibitory concentration (MIC) methods of antimicrobial susceptibility testing (AST) routinely used in the clinical laboratory. (**A**) Broth microdilution method, reprinted with permission from Heather Maughan (**B**) Kirby-Bauer Disk Diffusion Assay, reprinted with permission from Pearson Higher Education [2,21] (**B**).

While it is known that many disease conditions associated with chronic infections are often polymicrobial, polymicrobial communities are not currently assayed in the clinical microbiological model. Therefore, there is a large gap in the research literature on the effects of polymicrobial communities on the minimum inhibitory concentrations (MICs) of antibiotics and their relation to infectious processes and clinical outcomes [18]. The MIC can be defined as the lowest concentration of an antibiotic needed to prevent bacterial growth (bacteriostatic) or kill the bacterial population (bactericidal). MIC is currently determined in the clinical microbiological model through patient sampling and culture-dependent growth mechanisms. A patient sample is taken, which is used to inoculate several general, selective, and/or differential media (depending on the sampling site, specimen type, and expected organisms of that location), allowing for a variety of bacterial species to grow [19]. A clinical microbiologist then identifies the most likely causative agent of infection by visually identifying and distinguishing commensal and potentially pathogenic bacteria [19]. Bacterial colon(ies) are then selected for further examination and grown as pure culture on the appropriate media. In addition to speciation, antibiotic susceptibility testing (AST) is then performed in order to determine which antibiotic agent(s) convey the lowest MIC to eliminate the growth of the suspected pathogen (Figure 1) [19]. 

The two most common AST methods employed by the clinical microbiologist to determine MICs that are College of American Pathologists (CAP)-approved and regulated by the Clinical Laboratory Standards Institute (CLSI) are the broth microdilution and Kirby-Bauer Disk Diffusion assays [2]. The broth microdilution method utilizes a series of step-wise diluted antimicrobial agent(s) suspended in growth medium challenged against a standard concentration of unimicrobial bacterial inoculum [2]. After incubation (usually 16–24 h depending on the bacterial species), the lowest concentration of antimicrobial that was able to prevent bacterial growth (bactericidal or bacteriostatic) via turbidity observation is reported as the MIC [2] (Figure 1A). This is the most commonly employed AST method utilized in U.S. clinical laboratories, and most clinical laboratories have moved this methodology into an automated system such as the BD Pheonix^®^ or bioMérieux VITEK^®^. The Kirby-Bauer disk diffusion method uses antibiotic disks impregnated with standard drug concentrations that are placed on unimicrobial bacterial lawn of known cellular concentration plated on non-selective growth media [2]. Following incubation (usually 16–24 h depending on the species of bacteria), the radius of the zone of inhibition created by the antibiotic is measured to determine MIC [2,20,21] (Figure 1B).

It is worth noting that the CAP-approved and CLSI regulated AST methods (broth microdilution and Kirby-Bauer disk diffusion) are unimicrobial assays [2], and this testing method often identifies only one bacterial species as an infection’s causative agent. It is also important to note that only planktonic, or free-living, cells in suspension are evaluated via the broth microdilution AST assay, therefore ignoring the potential effects on antibiotic efficacy of biofilms. As the previous literature has stated, chronic infections are often polymicrobial in nature as well as biofilm-associated [22]; therefore, there is a critical disconnect between what research has shown to be important regarding antimicrobial efficacy and current clinical microbiological methods. 

Research scientists utilize the broth microdilution [23,24] and Kirby-Bauer disk diffusion assays to determine MIC as well as a variety of other methods such as direct contact testing (DCT) [25], LIVE/DEAD Viability/Cytotoxicity staining [26,27], quantitative polymerase chain reactions (qPCR) [28,29,30], pyrosequencing [31], and a variety of other in-house and/or non-standard methods [32,33,34,35,36]. In fact, much of the literature investigating changes to antimicrobial susceptibility in response to polymicrobial synergism investigated in this review were conducted with these research-specific AST methods rather than the CAP-approved and CLSI regulated AST methods. Because of this, clinical microbiologists are reluctant to accept this observed phenomenon orconduct clinical research into investigating polymicrobial interactions in AST determination, which has the potential to impact clinical care and patient prognoses. 

## 4. Mechanisms of Polymicrobial Synergism

**Figure 2:**
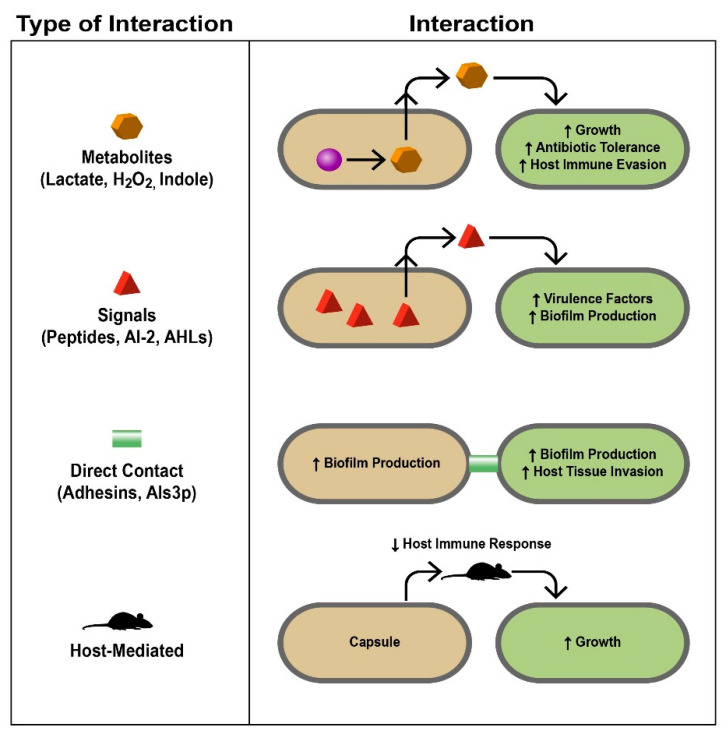
Mechanistic bases of polymicrobial interactions. Different interactions discussed in this review are summarized here. The left column lists the types of interactions and how they are mediated with specific examples, some of which are discussed in the text. The right column demonstrates how the interaction occurs and the response of the different microbes involved and the infected host. Signals, proteins, metabolites, and even the host immune system serve as liaisons between different microbes, allowing complex interactions to occur that impact the environments in which they live. During infection, these interactions ultimately lead to polymicrobial synergy and are therefore detrimental to the host (AI-2, autoinducer-2; AHLs, acyl-homoserine lactones). Taken from [37], reprinted with permission from *Journal of Microbiology* (Springer Nature).

There are many complex mechanisms pathogenic microbes can use to not simply survive, but also thrive in polymicrobial infections. Not only can pathogens work with each other, but they can work with commensal organisms, and even the host [37] (Figure 2). Many polymicrobial infections contain microorganisms that work cooperatively. 

### 4.1. Metabolites

In addition to biofilm formation, in which a hierarchical, often polymicrobial bacterial community can be established according to their nutrient and oxygen needs, metabolite cross-feeding, in which one species utilizes the metabolic pathway end-products of another species, allows for increased bacterial survival and growth This phenomenon has been extensively researched in oral bacteria, especially dental plaque [38]. An example of a cooperative relationship between bacteria is the mixed biofilm formed by *Veillonella atypica* and *S. gordonii*; *S. gordonii* produces lactate, which is then consumed by *V. atypica*; *S. gordonii* in turn responds to a diffusible molecule produced by *V. atypica* to produce amylase [38]. Ramsey et al. demonstrated that *Aggregatibacter actinomycetemcomitans* pathogenesis is enhanced when co-cultured with *S. gordonii*; *S. gordonii* produces L-lactate as its primary metabolite, which is then utilized by *A. actinomycetemcomitans* as a carbon source necessary for vigorous growth [39]. The fungus *Candida albicans* has been shown to possess a polymicrobial relationship with multiple bacterial species. It helps to create an anoxic environment allowing for gingival inflammation by the anaerobic bacteria *Porphyromonas gingivalis* [40]. Regarding metabolite cross-feeding, *C. albicans* metabolizes glucose produced by the bacteria *Streptococcus mutans* when it breaks down sucrose;this metabolization of glucose creates an acidic environment in which both the bacterial and fungal species thrive [40]. 

### 4.2. Signals

Quorum sensing, a mechanism of cell–cell communication based on population density mediated by signaling molecules, is another method of which microbes can benefit from polymicrobial infections. Microbial “cheaters” can use signals created by other microorganisms in order to avoid having to produce signals of their own. By listening in to the signals produced by other microbes (termed “eavesdropping”), these cheaters can perform tasks and react to environmental changes without having to expend energy on producing their own signals [41].

### 4.3. Direct Contact

Direct contact can contribute to polymicrobial synergy as microorganisms directly interact with each other. An example of this phenomenon is *S. epidermidis* protecting the fungus *C. albicans* from the antifungal fluconazole by producing an extracellular slime, increasing the content of their mixed biofilm and preventing contact of the antifungal drug with the fungus [42].

### 4.4. Host-Mediated

Host-mediated mechanisms of synergy are commonly seen in polymicrobial infections involving a virus, but can also be seen in polymicrobial infections involving bacteria and fungi. Host-mediated methods of synergism include mechanisms such as immune system modulation, in which a virus can decrease the host’s immune response, allowing for increased proliferation of other microbes. Often, other species in polymicrobial infections can benefit from this phenomenon, allowing for increased growth and more antibiotic resistance as the bacteria can focus on surviving the antibiotic instead of fighting the host’s immune system [43].

## 5. Polymicrobial Synergism and Its Impact on Antimicrobial Susceptibility

The literature surrounding the effect of polymicrobial synergism on antimicrobial susceptibility is, however, far from comprehensive. There are three main categories of these interactions investigated in this review—bacteria–bacteria, bacteria–fungus, and bacteria–viral, and each is less well elucidated than the last. The vast majority of the available literature is limited by two major factors—the limited number of species used in the experiments and the frequent use of non-standard conditions and models. Of all the polymicrobial combinations, the perhaps most commonly investigated relationship is the synergistic interaction of *P. aeruginosa* and *S. aureus*, two common colonizers of both the chronic wound and cystic fibrosis microbiomes. Polymicrobial interactions between bacteria–bacteria and bacteria–fungi with evidence of changes to antimicrobial efficacy are summarized in Table 1. 

### 5.1. Bacteria–Bacteria Interactions

Across a variety of experimental conditions and antibiotic classes, the co-culturing of *P. aeruginosa* (PA) and *S. aureus* (SA) has been shown to result in reduced antimicrobial susceptibility for *S. aureus.* Hoffman et al. [32], for example, demonstrated a doubling of the tobramycin MIC in SA when exposed to the PA exoproduct 4-hydroxy-2-heptylquinoline-*N*-oxide (HQNO). Orazi and O’Toole [33] exposed SA to the supernatant of a PA culture, and after testing some 240 antibiotics, found that “cell wall synthesis inhibitors and protein synthesis inhibitors…includ[ing] multiple representatives from the B-lactam, glycopeptide, aminoglycoside, macrolide, and tetracycline classes” showed decreased efficacy. Even as far back as 1978, Lebrun et al. [44] demonstrated a similar effect in a simple co-culture of the two species, with decreased susceptibility to rifamycin, vancomycin, penicillin, and cycloserine. While other studies had limited the direct exposure of PA and SA due to their competition in vitro, DeLeon et al. [34] used a wound-like media to allow for their co-culturing, which is consistent with the clinically observed conditions of several infectious processes, and also showed significant increases in tolerance for SA to both gentamicin and tetracycline. While this increase in tolerance to a wide variety of antibiotics has been more fully explored in this pair than that of other species, this is not, however, the only bacteria–bacteria interaction that has been studied. Vega et al. [35] demonstrated an increase in the tolerance of *S. typhimurium* to ciprofloxacin when exposed to the *E. coli* signaling molecules indole and tryptophan, and in a metabolically cross-feeding community, *E. coli* saw a doubling of MIC when exposed to tetracycline, even as the most susceptible organism in the culture [23]. Increases in tolerance to vancomycin were also observed in polymicrobial biofilm models using *Staphylococcus anginosus*, which were attributed to cell wall thickening, a mechanism which has been demonstrated elsewhere as an effect of synergistic interactions among bacteria [24]. Interactions between PA and anaerobes isolated from CF patient sputum have also been demonstrated to increase PA’s tolerance to piperacillin [29]. Though the largest body of research demonstrating changes in antimicrobial tolerance exists in this field, these interactions are certainly not limited to a single kingdom.

### 5.2. Bacteria–Fungus Interactions

Bacteria–fungus interactions, and their effect on polymicrobial susceptibility, are far less well examined than that of bacteria–bacteria interactions, but a body of research does exist. Of these, the relationship between *Candida albicans* and *S. aureus* has been examined in some detail, and along with increased biofilm formation [36] and an increase in synergistic lethality [40], an increased tolerance to both antibiotics and antifungal compounds has been observed when the organisms are in co-culture. Kean et al. [30] demonstrated a fourfold increase in tolerance to miconazole for *S. aureus* when cultured with *C. albicans*, an effect which has been supported by a similar decrease in *C. albicans* sensitivity to fluconazole when cultured with *S. mutans*. Examinations of the relationship between these organisms attributed the cause of the increased tolerance to a higher expression of drug efflux pumps on the part of *S. aureus*. In addition, the researchers demonstrated the ability of that same organism to respond to the quorum sensing molecule farnesol produced by *C. albicans* [27]. *S. epidermidis* has also been shown to have a protective effect on *C. albicans* by the production of an extracellular “slime”, which decreases the efficacy of fluconazole on the fungus [42]. Given that bacterial–fungal co-infection can occur across a wide number of body systems, including the enteric, respiratory, and oral cavity, this relative paucity of studies determining what changes occur in the respective organisms’ MICs demonstrates a gap in the available literature. 

### 5.3. Bacteria–Virus Interactions:

It is in this last category of interactions that the research is the most sparse, when focusing specifically on changes to antibiotic susceptibility. Though a variety of interactions between a number of respiratory viruses and bacteria [45,46], HIV and *Mycobacterium tuberculosis* [47], or the Epstein-Barr virus and *Porphyromonas endodontalis* or *P. gingivalis* have been described in detail [48], they have focused largely on the interactions occurring through the indirect mediation of host immune system activity, rather than a direct bacteria–virus interaction resulting in a change in MIC in constituent infectious bacteria. This trend is common in the available literature and viral–bacterial interaction groups. For instance, in a study of otitis media among pediatric patients, with one group having only a bacterial infection but the other having both bacterial and viral infections of the middle ear, differences in total clinical treatment success or failure were not statistically significant. However, a strong increase in the failure of initial antibiotic treatment was observed among the combined viral–bacterial infections, with 50% of the co-infected (bacterial and viral) group displaying failure compared to only 13% of the bacteria-only group [43]. In addition, of those in the bacteria-only failure group, 75% had bacteria with pre-existing resistance to the prescribed antibiotic, whereas 66% of the co-infected group’s bacteria were susceptible when evaluated using the Kirby-Bauer disk diffusion method [43]. This would seem to indicate that bacteria–virus co-infection is producing an effect on the antimicrobial susceptibility of the bacteria, but it is far less certain whether or not that effect relates more to the possible indirect immune suppression of the host due to the concomitant viral infection or due to the direct interactions of those two microorganisms with each other. It has been demonstrated that the presence of an Influenza A infection significantly decreases the physical penetration of antibiotics in a chinchilla model of otitis media, which may explain the number of failures of antibiotic treatment in that infectious process [49]. Outside of clinical studies, viruses have been shown to be able to increase the biofilm formation of *P. aeruginosa*, with a concomitant attendant effect on antimicrobial susceptibility [50], but it is less clear what effect the interaction of bacteria and viruses has, in a planktonic form, on antimicrobial susceptibility. In addition, the relevant literature has described direct bacteria–virus interactions within the context of enteric and respiratory infections, leading to increased bacterial and viral adherence and stability, which could suggest changes to bacterial MIC, although that has yet to be definitively determined [51,52,53,54]. Given the multiplicity of infections where viruses may be members of a polymicrobial community [50], this phenomenon certainly deserves more research. 

## 6. Discussion

There is a key disconnect between the fields of research and clinical microbiology. Even though it has been repeatedly demonstrated that biofilm-associated infections and/or a polymicrobial environment affect the susceptibility of bacteria to antibiotics, the current mode of AST determination does not reflect those conditions. Sadly, it has been extensively shown that patients harboring polymicrobial infections suffer worse outcomes across a wide variety of infectious processes. Polymicrobial infections were associated with higher frequencies of bacteremia than when patients were infected with *S. aureus* alone [55], with a “twofold higher risk of death than unimicrobial infection” [56] among adult patients, and a two and a third fold higher risk of mortality among neonates [57]. In sepsis, a more than threefold rise in mortality was observed among infants with polymicrobial bacteremia [58], and work relating the oral microbiota to the prognosis of CF patients showed that the presence of *S. aureus* and *P. aeruginosa* together (compared to unimicrobial infections with one or the other) in the mouth significantly impacted patient death rates [59]. In addition, the presence of *S. aureus* as a member in a polymicrobial infection has been demonstrated to independently increase patient morbidity and disease severity [4].

A longitudinal study of the mortality among CF patients showed that, though only 11% of those observed were co-colonized by methicillin-resistant *S. aureus* (MRSA) and *P. aeruginosa*, their mortality rate was 23%; over the same period of time, those patients without that co-colonization suffered only a 1% mortality rate [60]. Polymicrobial infections of post-traumatic osteomyelitis show similarly worse outcomes, with a fivefold higher rate of amputation, though a decreased rate of mortality in those cases [61]. It is important to note, however, that these treatments all used the standard unimicrobial AST methods for the selection of antibiotics. If the polymicrobial nature of these infections and those among patients with CF, chronic wounds, otitis media, and necrotizing fasciitis were taken into account, would patient outcomes be better? 

A few studies support an increase in positive outcomes when treatment modalities are changed to take into account the effects of a polymicrobial infection. Dowd et al. [62], using three different treatment modalities for chronic wounds, showed that when the constituent bacteria of the wounds were taken into account using NGS technologies (and therefore more likely to identify polymicrobial infections due to the elimination of culture-bias), the choice of systemic antibiotics changed to better assault these organisms, and wound closure rate increased by more than 25%, resulting in “improve[d] overall healing rates, reduce[d] healing times, and enhance[d] healing trajectories” compared to wounds evaluated using traditional culturing and identification methods. A retrospective study considering treatment of chronic wounds also demonstrated strong, positive changes in outcome for patients when “comprehensive molecular diagnostics” were used to guide antibiotic treatment choices, taking into consideration the effects of so-called “functional equivalent pathogroups”, the molecular diagnostic terminology for commonly identified co-infecting organisms within a polymicrobial infection [63]. 

It is entirely reasonable, therefore, to propose that the management of polymicrobial infections would be more efficacious if current AST methods were either supplemented by, or were modified to take into account, a polymicrobial assay method for infections that are notoriously polymicrobial. This change would allow for clinical microbiology to integrate its best practice model with the body of research that academic microbiologists have produced, and we hypothesize that this could lead to increases in treatment efficacy, promoting positive patient prognoses and potentially leading to decreased rates of mortality and morbidity across a wide variety of pernicious conditions currently affecting populations globally.

## 7. Materials and Methods 

Literature review for material in this publication was accomplished using the NCBI PubMed database and relevant search criteria for each subsection. For those sections detailing interactions between various kingdoms, articles were excluded if they did not include data on changes to minimum inhibitory concentration of the organisms studied or showed comparison of outcomes in clinical settings. Common terms used in those searches include “MIC”, “minimum inhibitory concentration”, “antibiotic”, “susceptibility”, “interaction”, and “synergism.” Articles were also prioritized for inclusion if they were more recently published or used CLSI-regulated and/or CAP-approved assays to determine changes to constituent minimum inhibitory concentration. This was performed to ensure that the articles were pertinent to the overall topics focused on in this work, and that they were relevant to possible translational work for clinical microbiologists. When relevant to the material included, and due to the scope of this review, where appropriate, literature reviews of specific interactions between pairs of organisms and infectious agents were used. While many other outcomes of synergistic effects of polymicrobial interactions have been investigated in the literature, including virulence and virulence factor production and biofilm formation, studies specifically investigating changes to antimicrobial susceptibility were selected for inclusion in this review. 

## Figures and Tables

**Table 1:** Summarized selection of changes to antimicrobial efficacy in polymicrobial conditions. This figure summarizes the observed effects of a polymicrobial condition on antibiotic efficacy in both bacteria–bacteria and bacteria–fungus interactions across a variety of species and antimicrobial combinations.

Citation	Organisms Studied	Observation
***Bacteria–Bacteria***		
Hoffman et al. [32]	*P. aeruginosa*, *S. aureus*	2x increase in tolerance to tobramycin
Orazi and O’Toole [33]	*P. aeruginosa*, *S. aureus*	Increased tolerance to B-lactam, glycopeptide, aminoglycoside, macrolide, tetracycline classes
Lebrun et al. [44]	*P. aeruginosa*, *S. aureus*	Increased tolerance to rifamycin, vancomycin, penicillin, cycloserine
DeLeon et al. [34]	*P. aeruginosa*, *S. aureus*	Increased tolerance to gentamicin, tetracycline
Vega et al. [35]	*E. coli*, *S. typhimurium*	Increased tolerance to ciprofloxacin
Adamowicz et al. [23]	*E. coli*, *S. typhimurium*	Increased tolerance to tetracycline
Tavernier et al. [24]	*P. aeruginosa*, *S. aureus*, *S. typhimurium*	Increased tolerance to vancomycin
***Bacteria–Fungus***		
Harriot et al. [36]	*C. albicans*, *S. aureus*	Increase in biofilm formation
Todd et al. [40]	*C. albicans*, *S. aureus*	Lethality increase
Kean et al. [30]	*C. albicans*, *S. aureus*	4x increased tolerance to miconazole
Kong et al. [27]	*C. albicans*, *S. mutans*	Increased tolerance to fluconazole, response to farnesol
Förster et al. [42]	*C. albicans*, *S. epidermidis*	Increased tolerance to fluconazole, “slime” production
